# Case of Excision of the Uterus

**Published:** 1850-11

**Authors:** Paul F. Eve

**Affiliations:** Prof. of Surgery in Med. College of Georgia


Art. II.—*Case of Excision of the Uterus.* By Paul F. Eve, M. D., Prof. of Surgery in Med. Coll. of Georgia. With Remarks, by C. D. Meigs, M. D., Prof. of Obstetrics and Diseases of Women and Children in Jefferson Med. Coll. of Philadelphia.

*To the Editor:* When Prof. Eve, of Augusta, Ga., passed through Philadelphia, on his return from the meeting of the Association at Cincinnati, he gave me a pathological specimen, which is now in my museum. This specimen consists of the uterus of a woman of color, which was removed by Professor Eve, in the hope that, by such a desperate operation, he might be able to rescue the patient from the imminent death which seemed by no other means to be avoided. The uterus, which he removed in the manner described in his letter, has been very much changed in its external form by the ravages of cauliflower excrescence.

I do not know that any American surgeon has heretofore extirpated the entire uterus *in situ*—an operation that is said to have been first performed by M. Sauter, of Constance, in 1822.

M. Colombat de l'Isèru informs us that the operation has been executed by Sauter, by Hoelscher, twice by Siebold, and thrice by Langenbeck; four times by Blundell; once by Bauner; once by M. Lizars; twice by Récamier; once by Dubled; twice by Reux, and once by M. Delpech; while this operation by Prof. Eve adds one integer to the whole number, which amounts to twenty operations, in all of which the result was contrary to the hopes of surgeons.

M. Colombat expresses the opinion that operations for the removal of the womb *in situ* ought not to be in future performed, in consequence of the disastrous summing up of the statistical records. He does not apply his objections to the cases of incurable inversion of the organ.

There are too many examples of recovery after extirpation of the inverted organ to leave any doubt on the mind as to the hopefulness of such an operation. Still, as I have firm confidence in the opinions I have published in other places, as to the power of spontaneous cure of inversio uteri, I should hesitate long before resorting to the measure of extirpation. In my friend's operation, there is cause to congratulate him upon the skill and resolution manifested by him, and upon the very hopeful success up to a certain point.

The following extract, from Prof. Eve's communications, will show that, but for the recommencement of the original heterologue development in the vagina, the patient had, in the most remarkable manner, been rescued from death.

I send you herewith an extract of a letter from Prof. P. F. Eve; also, a letter from Dr. J. A. Eve; and lastly, extracts from two letters from the surgeon.

Very respectfully, your obedient servant,

CH. D. MEIGS.

"On the 16th of April last, I removed the entire womb from a patient, who has recovered. The operation was performed at my surgical infirmary, in which I was assisted by my cousin, Dr. J. A. Eve, Professor of Obstetrics and Diseases of Women and Infants, and by Drs. Murray, H. Campbell, Longstreet, and Montgomery, and in the presence of several others connected with the profession.

"The patient is a negro woman, twenty-eight years of age; has been married, but never conceived, as she believes. For more than three years, she has been laboring under uterine affection; at least, she has been annoyed for about that length of time by a vaginal discharge. The history of diseases among our negro population is generally very imperfect and unsatisfactory; and this is especially true as regards uterine derangements. All we can obtain, in the present case, is, that the patient experienced great irregularity in menstruation, and had frequent hemorrhages from the vagina.

"Yours, &c., P. F. EVE."

We now refer to Dr. J. A. Eve's statement of the case, as he observed it before she arrived at the Infirmary, in Augusta.

AUGUSTA, April 24, 1850.

DR. P. F. EVE—Early on the morning of the 10th instant, I was called to visit Mary, the patient whose womb you extirpated on the 16th, in consultation with Drs. Murray and Cook, some eleven or twelve miles from the city.

Under the influence of morphine, which had been given before my arrival, the patient had become easy. On examination, I found a tumor of considerable size in the hypogastrium, and the whole pelvis, to the outlet, filled and blocked up with a lobulated, convoluted, incomprehensible mass, from which issued a copious and horribly fetid discharge.

As this was unquestionably carcinoma, cauliflower excrescence, encephaloid tumor, or some malignant growth, the patient's certain doom was death, after a few months, or at most a year, of miserable existence worse than death, unless rescued by surgery, in the performance of a heroic operation which would involve the removal of a portion or the whole of the uterus.

If such an operation would ever be indicated or warranted, the age, (twenty-eight years), the vigor of constitution, and the comparatively unimpaired health of the patient, made it proper in this case.

In consultation, I suggested to Drs. Murray and Cook that, as neither of us could take charge of, or do justice to her case, so far from our respective residences, she should be removed, as soon as practicable, to your infirmary, where she would enjoy every advantage and benefit that favorable circumstances, as well as science and art, could afford her case ; and that we should all meet and confer with you after her removal to this place ; to which suggestions these gentlemen cordially acceded.

I know nothing of the previous history of this case, except what has been related to us by Dr. Murray. In consultation, all the physicians present concurred in opinion with you, that the operation was one of extreme danger, and the probabilities were as many, perhaps, as a hundred to one against its success.

Before the operation, Dr. Murray and myself visited the patient, explained to her its great danger, and the very great probability that she might not survive it ; telling her that, although it afforded but little hope, it was the only hope of delivery from suffering and death. We told her, further, that it rested entirely with herself to determine whether or not she would submit to the operation. Without persuasion or influence of any kind, she determined promptly and unhesitatingly to submit to the operation, terrific as it was represented to be. She is now doing well, and in all probability wlll return home next week.

Your sincere friend,

J. A. EVE.

*Operation.*—The bowels having been previously emptied, a large quantity of urine was drawn off by the catheter, which diminished considerably the hypogastric tumor, and proved the bladder to have been generally distended, as there was no urgency to micturition—in fact, the patient was unconscious of distension. About two pints were thus evacuated. Chloroform was now inhaled to its full anæsthetic effects, when the vaginal tumor was seized by various forceps, but which, after large tubercular masses were torn off, was finally brought down to the os externum by the left hand. Finding it impossible to remove the firm resisting body now presented to view, it was carefully excised from above downwards, or in an antero-posterior direction, by the knife—I confess, with some suspicions at the time, it might be the uterus. One artery (now believed to be the left uterine), throwing out blood quite vigorously, was seized, and an animal ligature cast around it. A solution of sulphate of zinc was applied to restrain further hemorrhage, which had been considerable.

There was no protrusion of the bowels, nor was the case followed by any very severe symptoms. A most rigid confinement to the horizontal position was strictly enforced for about ten days, with absolute diet, &c., &c. The bladder, it is presumed, filling up again, pushed the intestines backwards, while the opening made into the peritoneum was closed by agglutination and subsequent adhesion. The rectum was evacuated on the fourth day after the operation by warm water, and the bowels were moved freely by oil on the fifth.

In the mass removed, the uterus is readily recognized, with its Fallopian tubes, broad and round ligaments; but the os tincæ is involved in the encephaloid degeneration. The tumor in the vagina was about the size of a child's head at full term. No one, it is believed, who has examined it, has entertained the least doubt but that the entire womb was removed, and this includes, besides the gentlemen who witnessed the operation, Dr. R. D. Mussey, Professor of Surgery in the Medical College of Ohio, and Chairman of the Committee on Surgery for the past year in the American Medical Association; and my preceptor, Dr. C. D. Meigs, the distinguished Professor of Obstetrics, &c., &c., in the Jefferson Medical College, with whom the uterus has been deposited, and who has kindly insisted upon presenting the case to the profession in his his own way.

During my absence at the meeting of the Medical Association in Cincinnati, the case was left under the care of my relative and assistant, Dr. A. P. Longstreet. The patient returned home on the 3rd of May, visited Augusta again on the 20th, to inquire why she had had no hemorrhage (menstruation) since the operation; and, in answer to a letter, Dr. Murray writes, on the 10th of June, that he saw her "up and about" the day before, and promised to bring her, in a few days, to my office.

Fifteenth of June, two months after the operation, the patient, Mary, has called, after riding eleven miles on a loaded lumber wagon. She is much improved in flesh and appearance, and has enjoyed good health. She says there has been a slight show of blood but once since the operation, and only a moderate discharge at times of colorless fluid. But I regret to add, we have most unmistakable evidence, both ocular and by touch, of a rapid reproduction of the encephaloid disease, which in all probability must sooner or later destroy life.

Extract of a letter, dated—

AUGUSTA, July 29th, 1840.

*My Dear Doctor*—I write to say Mary, my *non-uterine patient, is dead.* She died on the 22d of July, having lived three months and a week after the operation. She became œdematous, (ascites, also), but had no hemorrhage, neither protrusion of the disease from the os externum. I regret no post-mortem was made by the physician in attendance, and I only learned her decease incidentally at the time. PAUL F. EVE.

DR. C. D. MEIGS. [*Am. Jour. Med. Sci.*

